# The RNA binding protein HuR differentially regulates unique subsets of mRNAs in estrogen receptor negative and estrogen receptor positive breast cancer

**DOI:** 10.1186/1471-2407-10-126

**Published:** 2010-04-06

**Authors:** Robert Calaluce, Matthew M Gubin, J Wade Davis, Joseph D Magee, Jing Chen, Yuki Kuwano, Myriam Gorospe, Ulus Atasoy

**Affiliations:** 1 Departments of Surgery, University of Missouri, One Hospital Drive, Columbia, Missouri 65212, USA; 2 Health Management and Informatics, University of Missouri, 187 Galena Hall, Columbia, Missouri 65212, USA; 3 Statistics, University of Missouri, 146 Middlebush Hall, Columbia, Missouri 65211, USA; 4 Molecular Microbiology and Immunology, One Hospital Drive, Columbia, Missouri 65212, USA; 5 Child Health, One Hospital Drive, University of Missouri, Columbia, Missouri 65212, USA; 6 National Institute of Aging, 251 Bayview Boulevard, Baltimore, Maryland 21224, USA

## Abstract

**Background:**

The discordance between steady-state levels of mRNAs and protein has been attributed to posttranscriptional control mechanisms affecting mRNA stability and translation. Traditional methods of genome wide microarray analysis, profiling steady-state levels of mRNA, may miss important mRNA targets owing to significant posttranscriptional gene regulation by RNA binding proteins (RBPs).

**Methods:**

The ribonomic approach, utilizing RNA immunoprecipitation hybridized to microarray (RIP-Chip), provides global identification of putative endogenous mRNA targets of different RBPs. HuR is an RBP that binds to the AU-rich elements (ARE) of labile mRNAs, such as proto-oncogenes, facilitating their translation into protein. HuR has been shown to play a role in cancer progression and elevated levels of cytoplasmic HuR directly correlate with increased invasiveness and poor prognosis for many cancers, including those of the breast. HuR has been described to control genes in several of the acquired capabilities of cancer and has been hypothesized to be a tumor-maintenance gene, allowing for cancers to proliferate once they are established.

**Results:**

We used HuR RIP-Chip as a comprehensive and systematic method to survey breast cancer target genes in both MCF-7 (estrogen receptor positive, ER+) and MDA-MB-231 (estrogen receptor negative, ER-) breast cancer cell lines. We identified unique subsets of HuR-associated mRNAs found individually or in both cell types. Two novel HuR targets, *CD9 *and *CALM2 *mRNAs, were identified and validated by quantitative RT-PCR and biotin pull-down analysis.

**Conclusion:**

This is the first report of a side-by-side genome-wide comparison of HuR-associated targets in wild type ER+ and ER- breast cancer. We found distinct, differentially expressed subsets of cancer related genes in ER+ and ER- breast cancer cell lines, and noted that the differential regulation of two cancer-related genes by HuR was contingent upon the cellular environment.

## Background

Over the past decade array technologies have provided several new means for profiling global changes in gene expression. The power of DNA microarrays is perhaps best illustrated in the way it has been used to differentiate treatment responses in patient populations. Individualized and targeted therapy for several tumors, based upon underlying differences at the molecular level among gene expression profiles, is beginning to replace the traditional morphological-based treatment paradigm [1-3]. Genome wide microarray analyses, however, are inherently flawed since they globally profile the steady-state levels of mRNA, referred to as the transcriptome. Cellular protein expression levels, however, do not directly correlate with steady-state levels of mRNAs. It is well accepted in the RNA field that there is a poor correlation between steady-state RNA levels and protein. This discordance has been attributed to posttranscriptional control mechanisms affecting mRNA stability and translation. Steady-state mRNA levels of genes, controlled partially or totally at this level, may be misleading. Gygi and colleagues have shown that correlation between mRNA and protein levels could not be predicted from only mRNA steady-state levels [4]. They observed that some genes had the same mRNA levels but protein levels varied more than 20 fold. Conversely, some proteins were of equal expression but their respective mRNA level varied by more than 30-fold. They concluded that "transcript levels provide little predictive value with respect to the extent of protein expression" [4]. Additionally, Idekar and colleagues have described similar results for the galactose gene [5].

Although our understanding of transcriptional gene regulation is advanced, posttranscriptional gene regulation remains largely unexplored. It is becoming clear, however, that this is an important mode of gene regulation, especially for proinflammatory genes. These genes appear to be posttranscriptionally regulated by RNA binding proteins (RBPs) which interact with AU-rich elements (AREs) in the 3' untranslated region (UTR) of mRNAs. Approximately 3,000 human genes contain AREs, representing 8% of the human genome [6]. Many of these genes which possess AREs are in areas of transient biological responses, including cell growth and differentiation, immune responses, signal transduction, transcriptional and translational control, hematopoiesis, apoptosis, nutrient transport, and metabolism [6,7].

New methodologies have provided global identification of *in vivo *mRNA targets of different RBPs. One of these, termed the ribonomic approach, involves the immunoprecipitation of ribonucleoprotein complexes (RNPs) with antibodies against different RBPs, extraction of mRNA, and hybridization to microarrays [8-10]. This approach, also referred to as RIP-Chip, enables investigators to identify groups of posttranscriptionally regulated mRNAs coordinately controlled by RBPs during various biological processes. A new paradigm, the posttranscriptional operon hypothesis, has been developed which states that RBPs coordinately regulate the expression of biologically related molecules [11,12]. This paradigm is being confirmed by the work of many different laboratories as our understanding of posttranscriptional regulation broadens and putative operons are described [8,13-17]. HuR is an RBP that binds to AREs of many proto-oncogenes and labile mRNAs. It has emerged as a key regulatory factor which stabilizes and translationally enhances its targets mRNAs, and affects their transport from the nucleus to the cytoplasm [18-20]. HuR belongs to the *ELAV *(*embryonic lethal abnormal vision*) family found in mammalian cells containing four members: HuR, HuB, HuC, and HuD. HuR is the only ubiquitously expressed member. The other family members are found primarily in the central nervous system and gonadal tissue [18]. Many HuR targets are cytokines, chemokines, and other early-response genes [21,22].

Of the hallmarks of cancer originally described by Hanahan and Weinberg, HuR has been demonstrated to control expression of genes in multiple areas of malignant transformation [23]. Consequently, HuR has been suggested to function as a tumor maintenance gene, permissive for malignant transformation, tumor growth, and perhaps metastasis [24]. HuR has been described in the literature as controlling the expression of many cancer-relevant genes, including those that encode these proteins: Prothymosin-α, Bcl-2, Mcl-1, SirT1, TGF-β, MMP-9, MTC-1, uPA, VEGF-α, HIF1-α and cyclins A1 (CCN A1), B1 and D1 [25-35]. Increased levels of HuR have been associated with a more aggressive breast cancer and a worse prognosis [36-38]. Of significance, HuR has been described as posttranscriptionally regulating the expression of many breast cancer relevant genes including those that encode Glut-1, ERα, COX-2, IL-8, Cyclin E1, and most recently BRCA-1 [36,39-44]. HuR RIP-Chip analysis has recently identified Thrombospondin 1 as a key HuR target in the MCT-1 transformed estrogen receptor positive (ER+) cell line MCF-7 [45]. Its interactions, however, are complex and, at times, HuR may interact with miRNAs such as Let-7 to translationally suppress the expression of *C-MYC *mRNA [46].

Since HuR has been described as regulating the expression of many cancer relevant genes, we asked whether it may coordinately regulate breast cancer genes in ER+ and ER- breast cancer. We performed a HuR RIP-Chip analysis on MDA-MB-231 (ER-) and MCF-7 (ER+) cell lines to identify cancer-relevant genes, not known to be regulated by HuR, and potential novel breast cancer targets. Our studies indicated that HuR was associated with unique subsets of mRNAs in each cell line as well as a subset of HuR associated mRNA targets common to both. We chose two cancer-associated genes, *CD9 *and *CALMODULIN 2 *(*CALM2*), highly expressed in both cell lines, and functionally validated the role of HuR in regulating their expression. Unexpectedly, HuR differentially regulated the same target, *CD9*, in both cell lines in an opposite manner. Moreover, we found presumptive differential regulation of *CALM2 *by HuR, as HuR interacted only with *CALM2 *mRNA, but not with family members *CALM1 *and *CALM3 *mRNAs. We discovered that HuR interacts with many breast cancer-relevant genes not previously known to be controlled by HuR, and target genes which have not been shown to be cancer related. This latter category may indeed represent novel cancer genes discovered by HuR RIP-Chip analysis.

## Methods

### Cells in culture

The MDA-MB-231 (MB-231) and MCF-7 cell lines were obtained from American Type Culture Collection (Manassas, VA). The cell lines were maintained at 37°C in a humidified atmosphere of 95% air and 5% CO_2_. MB-231 cells were grown in RPMI (GIBCO^®^, Invitrogen™, Carlsbad, CA) containing 10% fetal calf serum (Hyclone, Thermo Fisher Scientific, Waltham, MA), 0.5 mM L-glutamine (GIBCO^®^), 25 mg/ml glucose (Sigma-Aldrich), HEPES (GIBCO^®^) and sodium pyruvate (GIBCO^®^). MCF-7 cells were grown in DMEM (GIBCO^®^) supplemented with 10% fetal calf serum.

### HuR Immunoprecipitations (RIP-Chip)

HuR RIP-Chip was performed as previously described [8,47,48]. Briefly, lysates were prepared from exponentially growing MB-231 and MCF-7 cells. Equal amounts of protein lysates were used (100-300 μg). HuR monoclonal antibody 3A2 (made in our laboratory from the 3A2 hybridoma, generously provided by Dr. Joan Steitz, Yale University, New Haven, CT), or isotype control IgG1 (BD Biosciences, San Jose, CA), were pre-coated onto Protein A Sepharose beads (PAS) and extensively washed. Lysates from each cell initially were pre-absorbed with 30 μg of IgG1 and then removed by addition of PAS beads. Individual pull-downs were performed at 4°C for only 1-2 hr to minimize potential re-assortment of mRNAs.

### RNA amplification

The entire amount of recovered RNA per immunoprecipitation was amplified using the WT-Ovation™ Pico RNA Amplification System protocol (NuGen, San Carlos, CA). Forty ng of total RNA was used as starting material to generate at least 6 μg of cDNA. Amplified cDNA was purified using Zymo Research Clean and Concentrator™-25 (Zymo Research, Orange, CA). Three μg of amplified and purified cDNA was incubated at 50°C for 30 minutes with 5 μl of UNG buffer and 5 μl UNG enzyme and 60 minutes with 5 μl labeling buffer and 5 μl ARP (biotin) solution as described in NuGen's labeling protocol for the Illumina BeadArray platform. All samples (total RNA, amplified cDNA, and biotin labeled amplified cDNA) were quantitated using a Nanodrop™ (Thermo Fisher Scientific, Waltham, MA) spectrophotometer. RNA quality and integrity were assessed on selected samples with the Experion™ automated electrophoresis system (Bio-Rad, Hercules, CA).

### Microarray

Biotin-labeled, amplified cDNA (1.5 μg) was hybridized to a Sentrix^® ^Human-6 v.2 Whole Genome Expression BeadChips (Sentrix Human WG-6; Illumina, San Diego, CA). Each chip tested 6 samples and contained 47,293 gene targets, representing 18,025 distinct RefSeq genes that are not pseudogenes. A total of 3 chips were used for this experiment. The chips were hybridized at 48°C for 20 hr in the hybridization buffer provided by the manufacturer. After hybridization, the chips were washed and stained with streptavidin-C3. The chips were scanned on the BeadArray Reader, as described by Illumina at http://www.illumina.com. The Illumina BeadStudio software was used to assess fluorescent hybridization signals.

### Quantitative RT-PCR

Selected genes were validated by quantitative RT-PCR. Briefly, cDNA was generated from the same samples as previously described for the microarray experiments using 10 ng total RNA and the SuperScript™ III Platinum^® ^Two-Step qRT-PCR Kit with SYBR^® ^Green (Invitrogen Carlsbad, CA). RT-PCR was performed on the StepOne™ Real-Time PCR System (Applied Biosystems, Foster City, CA). Each sample was run in triplicate for these genes and the cDNA was divided equally per reaction in a 20 μl volume. The PCR conditions were: 50°C for 2 minutes and 95°C for 2 minutes, followed by 40 cycles of 95°C for 15 seconds alternating with 60°C for 30 seconds. Melting curve analysis was performed on every reaction to confirm a single amplicon. For each cell line, differences in gene expression were determined using the equation 2^-ΔΔCt^, where the *C*_*t *_value for either the HuR or IgG IP was subtracted from the *C*_*t *_value of the *GAPDH *control to yield the Δ*C*_*t *_value. For each cell line, the Δ*C*_*t *_value for the HuR and IgG IP were computed in triplicate and averaged to give one ΔΔ*C*_*t *_value per sample. Primers used:

Human RT *GAPDH *Forward 5' AGCCTCAAGATCATCAGCAATGCC 3'

Reverse 5' TGTGGTCATGAGTCCTTCCACGAT 3'

Human RT *HuR *Forward 5' ATGAAGACCACATGGCCGAAGACT 3'

Reverse 5' AGTTCACAAAGCCATAGCCCAAGC 3'

Human RT *CD9 *Forward 5' TCAGACCAAGAGCATCTTCGAGCA 3'

Reverse 5' ACCAAGAGGAAGCCGAAGAACAGT 3'

Human RT *CALM2 *Forward 5' CTGACCAACTGACTGAAGAGCAGA 3'

Reverse 5' TTCTGTGGGATTCTGCCCAAGAG 3'

### Cloning strategy of HA HuR

Hemagglutinin (HA) tagged human HuR was cloned into the *NheI *and *XhoI *sites of the pZeoSV2 (-) vector (Invitrogen). The plasmids were sequenced in both directions to confirm identity. Cells were transfected with either pZeo HA HuR or pZeo empty vector using Lipofectamine 2000 (Invitrogen). After five days transfected media was removed and replaced with fresh medium containing 200 μg/ml of Zeocin antibiotic (Invitrogen). Cells were selected for a ten day period. After ten days, the selected cells were maintained in 50 μg/ml of Zeocin to maintain pZeo HA HuR and empty vector expression. No viable cells remained in the untransfected well. Cells were then cloned by limiting dilution.

### Lentiviral RNAi HuR knock-down

In order to knockdown HuR, PSICOOLIGOMAKER v1.5 http://web.mit.edu/ccr/labs/jacks/was used to identify optimal shRNAs sequences to HuR. We tested multiple sequences and chose GGATCCTCTGGCAGATGT, identified and designated shRNA H760. Annealed sense and antisense DNA (Integrated DNA Technologies, Inc, IDT, Coralville, IA), along with stem loops to create hairpin, were cloned into the *HpaI *and *XhoI *restriction sites in the Lentilox pll3.7 vector (ATCC). After sequence verification, lentivirus was packaged in 293FT cells using ViraPower™ Lentiviral Expression Systems (Invitrogen) following manufacturer's protocol. Both MB-231 and MCF-7 cells were seeded at a density of 100,000 cells in 100 mm tissue culture plates with 10 ml of media. The following day lentivirus, expressing either GFP and no shRNA (empty lentilox control) or GFP and HuR shRNA H760, was added at a multiplicity of infection (MOI) of 10 along with polybrene (8 μg/ml) (Sigma-Aldrich Corp, St. Louis, MO). After five days, cells were harvested by trypsinization and sorted for GFP expression using BD FACSDiva (BD Bioscience). Cells were cloned by limiting dilution and GFP expression was assessed using FACScan (BD Bioscience) and CellQuest software (BD Bioscience). GFP expression was >98% and indicated homogenous cell population.

### SDS-PAGE and Western Blot Analysis

Western analysis was performed as described previously with slight modifications [47]. Briefly, cells were harvested and lysed in triple-detergent RIPA buffer with protease inhibitor cocktail (Roche, Pleasanton, CA). For nuclear and cytoplasmic fractionation, the NE-PER kit was used (Pierce, Rockford, IL). Protein quantity was determined by Bradford Assay. Forty μg of protein was electrophoresed on a 12% SDS-polyacrylamide gel and transferred to a nitrocellulose membrane. The membrane was blocked with 5% nonfat milk at room temperature for 1 hr and incubated with anti-β-tubulin (1 μg/ml, Sigma-Aldrich) at 4°C overnight. After washing, the membrane was incubated with monoclonal anti-HuR clone 3A2 antibody (1 μg/ml) at room temperature for 1 hr, or anti-CD9 antibody (1:100) (Santa Cruz Biotechnology, Inc., Santa Cruz, CA) at 4°C overnight. The secondary antibody used was sheep anti-mouse Ig horse radish peroxidase (1:4000) (GE Healthcare, Piscataway, NJ), incubated at room temperature for 1 hr. Specific proteins were detected using chemiluminescence (GE Healthcare). HuR knock-down was determined to be >90% using Bio-Rad's Quantity One software (Bio-Rad) normalizing to β-tubulin, and HuR over-expression was quantitated in a similar manner.

### Biotin Pull-down

Biotinylated transcripts were synthesized using cDNA that was prepared from MB-231 cells. Templates were prepared using forward primers that contained the T7 RNA polymerase promoter sequence (CCAAGCTTCTAATACGACTCACTATAGGGAGA [T7]). Primers used for the preparation of biotinylated transcripts spanning the *CD9 *CR, and 3'UTR (NM_001769) and *CALM2 *CR and 3'UTR (NM_001743.3) were as follows:

*CD9 *CR 118-560: [T7] TCAAAGGAGGCACCAAGTGCAT and AACGCATAGTGGATGGCTTTCA

*CD9*3'UTR798-1231: [T7] AGTCAGCTTACATCCCTGAGCA and GACATTGTCATAATTTTTTATTATGTATC

*CALM2 *CR 72-515: [T7] GCTGACCAACTGACTGAAGA and CTTTGCTGTCATCATTTGTACAAA

*CALM2 *3'UTR 518-1128: [T7] AGACCTTGTACAGAATGTGTTAA and GGGTAAATTGTAATTTTTTTATTGGAA

*GAPDH *3'UTR: [T7] CCTCAACGACCACTTTGTCA and GGTTGAGCACAGGG TACTTTATT

The PCR-amplified fragments were purified and used as templates for *in vitro *synthesis of the corresponding biotinylated RNAs by MAXIscript kit (Ambion^®^, Applied Biosystems). Biotin pull-down assays were performed by incubating 40 μg of MB-231 cell lysates with equimolar of biotinylated transcripts for 1 hr at room temperature. The complexes were isolated using paramagnetic streptavidin-conjugated Dynabeads (Dynal^®^, Invitrogen), and bound proteins in the pull-down material were analyzed by Western blotting using an antibody recognizing HuR (Santa Cruz). After secondary-antibody incubations, the signals were visualized by chemiluminescence (Amersham Biosciences, GE Healthcare).

### Statistical Analysis of Microarray Data

Analysis of microarray gene expression data was primarily performed using the Linear Models for Microarray Data (limma) package [49] and the lumi package [50], available through the Bioconductor project [51] for use with R statistical software [52]. After data pre-processing was completed (Appendix), the statistical analysis was performed using moderated *t*-statistics applied to the log-transformed (base 2) normalized intensity for each gene using an Empirical Bayes approach [53]. Three contrasts of interest were computed and tested. The first was the difference between HuR pull-down and IgG background for the MB-231 cell line. Genes which exhibited significantly greater expression in the pull-down were considered to be in the HuR pellet for the MB-231 cell line. The second contrast was similar to the first, but for the MCF-7 cell line. The third and most important contrast was the difference between the first and second contrast, and can be viewed as a test of statistical interaction between HuR and cell line. For a given gene, this term can be interpreted as reflection of the synergistic relationship between HuR and estrogen in breast cancer. Adjustment for multiple testing was made using the false discovery rate (FDR) method of Benjamini and Hochberg [54] with an FDR of 10% as our cutoff for declaring significance. To facilitate interpretation, log fold changes were transformed back to fold change on the data scale (fluorescent intensity).

Gene ontology (GO) analyses were carried out on the list of significant genes based on the third contrast described above. The purpose of the analyses was to test the association between Gene Ontology Consortium categories [55] and differentially expressed HuR pellet genes between MB-231 and MCF-7. Using our defined gene universe (Appendix), GOstats [56] was used to carry out conditional hypergeometric tests. These tests exploit the hierarchical nature of the relationships among the GO terms for conditioning [57]. We carried out GO analyses for over-representation of biological process (BP), molecular function (MF), and cellular component (CC) ontologies, and computed the nominal hypergeometric probability for each GO category. These results were used to assess whether the number of selected genes associated with a given term was larger than expected under the null hypothesis, and a *p*-value cutoff of 0.01 was used. GO categories containing less than 10 genes from our gene universe were not considered to be reliable indicators, and are not reported.

## Results

### HuR immunoprecipitation from ER+ and ER- breast cancer cell lines

**Figure 1:**
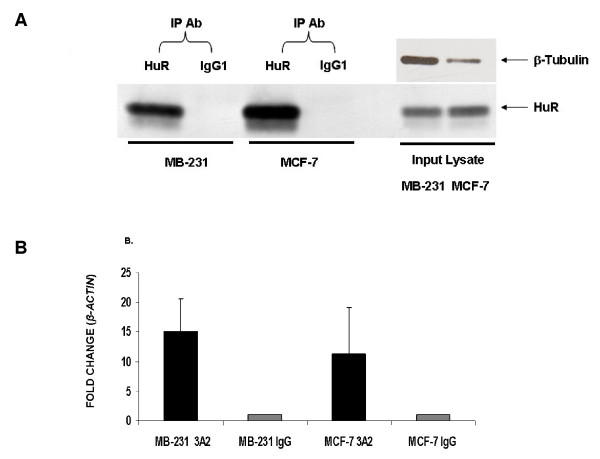
**Immunoprecipitation and RIP in MB-231 and MCF-7 breast cancer cells**. Immunoprecipitations were performed from MB-231 or MCF-7 cell lysates using anti-HuR monoclonal antibody (3A2) and IgG1 isotype control. **A**. IP Western of HuR revealed expected size band as detected by 3A2. Panel on right reveals amounts of HuR in lysates used from both cell lines. **B**. Verification by quantitative RT-PCR showed fifteen and eleven fold enrichments of *B-ACTIN*, a known HuR target, in the 3A2 IPs from MB231 and MCF-7, respectively. All ΔΔC_T _values were normalized to *GAPDH*. Experiments were done in duplicate (n = 2).

We first determined HuR protein expression levels in breast cancer cell lines. HuR is expressed in both the ER- and the ER+ cell lines, MB-231 and MCF-7, respectively (Figure 1A). RNA immunoprecipitation, using HuR monoclonal antibody 3A2, recovered HuR (Figure 1A) and revealed, by quantitative RT-PCR, a significant enrichment of up to fifteen fold for a known HuR target, *β-ACTIN *mRNA, as compared to isotype control (IgG1) and normalized to a non-target, *GAPDH *mRNA (Figure 1B). These data showed that HuR RIP specifically immunoprecipitate HuR protein and associated mRNAs, though absolute quantitative conclusions cannot be drawn since different amounts of lysates were used and efficiency of immunoprecipitation from different cell lines may differ.

### RIP-Chip from ER+ and ER- breast cancer cell lines identifies unique sets of associated mRNAs

**Figure 2:**
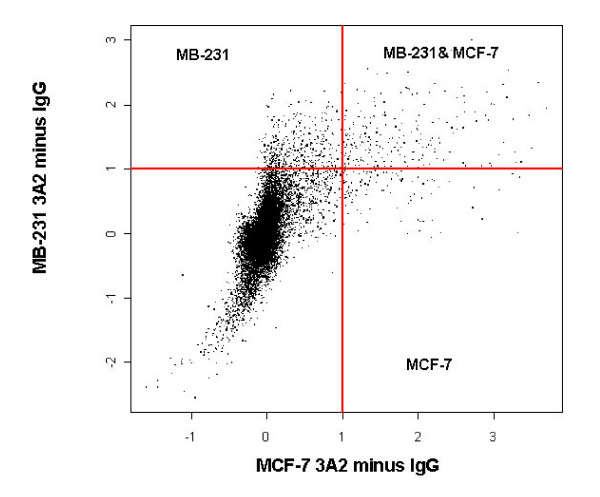
**HuR RIP-CHIP identifies distinct genetic profiles in ER+ and ER- breast cancer cells**. HuR immunoprecipitations were performed from MB-231 or MCF-7 cell lysates using HuR antibody and IgG1 isotype control hybridized to Illumina Sentrix arrays (47,000 genes). Control signals were subtracted. Results represent cumulative data from 12 different arrays. Experiments were done in triplicate (n = 3) for each cell line with matching controls. Scales are log_2_.

RIP-Chip was performed on cytoplasmic lysates from both breast cancer cell lines with HuR antibody and isotype control in order to determine HuR associated mRNAs. Each immunoprecipitation was done at least three independent times with matching controls. Signals from isotype control were subtracted out. Recovered mRNA was amplified and hybridized to Illumina Sentrix Human arrays consisting of 47,000 genes. Figure 2 represents a composite array generated by combining hybridizations to twelve different arrays (log_2 _scale). Three groups of HuR-associated target genes were identified: MB-231 targets in the left upper quadrant; both MB-231 and MCF-7 targets in the right upper quadrant; MCF-7 targets in the right lower quadrant. As expected, most of the mRNAs did not associate with HuR and were located in the lower left quadrant. There were 395 and 64 annotated genes, at least 2 fold or more enriched, associated with either MB-231 or MCF-7 cells, respectively, and 182 genes associated with both cell lines. A complete list can be found in Additional File 1, Figure S2. The raw data files are available in the NCBI database at the following link: http://www.ncbi.nlm.nih.gov/geo/query/acc.cgi?token=pdsnrqmiawukqlm&acc=GSE17820, NCBI Accession number GSE17820). These genes generally fell into three groups. Group 1 consisted of cancer-associated genes which were known HuR targets, such as *PTMA *mRNA. Group 2 consisted of genes which played a role in cancer but were not known to be HuR targets. Group 3 consisted of genes with an unknown function in cancer, but which may be regulated by HuR. These data revealed that HuR was associated with distinct subsets of mRNAs in ER+ and ER- breast cancer cells.

**Figure 3:**
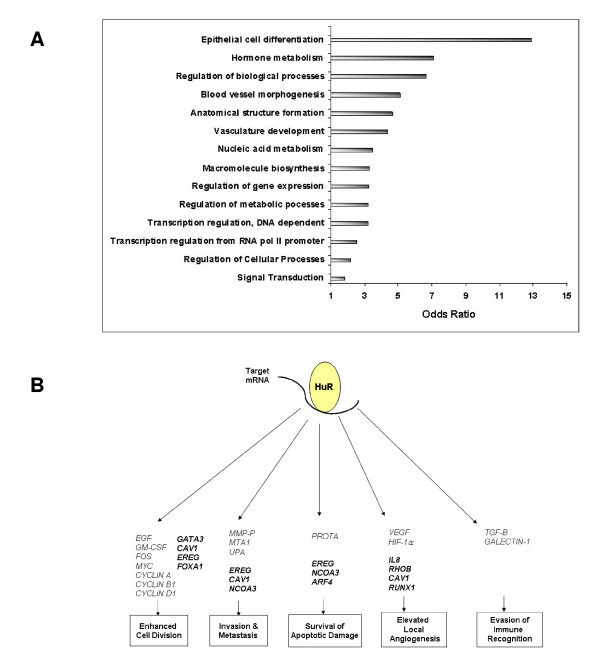
**GO Classification of genes found by RIP CHIP of potential HuR targets and their relationship to the Acquired Capabilities of Cancer Model**. **A**. Differentially expressed genes which are more represented in the Biological Processes (BP) GO category than expected. **B**. Original representation showing subsets of transcripts found to be targets of association with HuR (normal type). New transcripts found in this study with RIP-Chip (bold type). Enhanced expression upon binding to HuR influences several of the acquired capabilities of cancer cells described by Hanahan and Weinberg [23,24].

Gene Ontology (GO) analyses of differentially expressed significant genes between ER+ and ER- cells were categorized into Biological Process (BP), Cellular Component (CC), and Molecular Function (MF). GO analyses allow for the identification of gene families that may play significant roles related to these categories in expression profiles. Most of the differentially expressed genes (155) were found to be more abundant than expected in 14 BP categories (Figure 3A). Three MF categories consisted of 100 genes with most of these (83) related to protein binding and transcription activator activity. The CC categories contained the least (34) and were primarily associated with the Golgi apparatus. For the complete GO analyses see Additional File 2, Table S1. In Table S1 we list the top HuR associated mRNAs in the different categories which were approximately 5 fold enriched or greater. As can be seen in Figure 3B, a partial listing of some of these genes (in bold) are candidate members to multiple areas of cancer control, as suggested by Hanahan and Weinberg. We note that though β-*ACTIN *mRNA was amongst the most abundant of HuR-associated mRNAs in MCF-7 cells, β-*ACTIN *mRNA levels were only 3.93-fold higher in HuR IP compared to IgG IP in MB-231 cells. Therefore, since this was less than the 5-fold cut-off we employed for Table S1, it is not listed. Thus, these results may have identified novel HuR-controlled genes which may play roles in breast carcinogenesis in a cancer subtype-specific fashion.

### Validation of HuR targets CD9 and CALM2 by real-time PCR and biotin pull-down analyses

**Figure 4:**
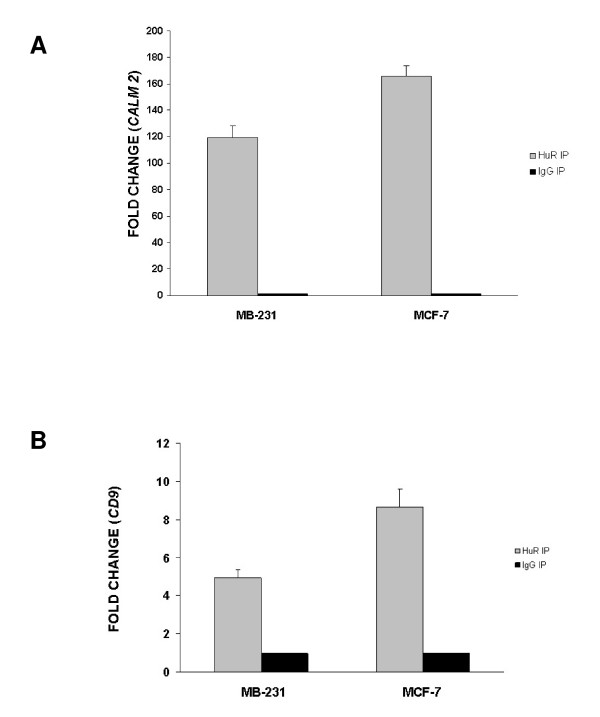
**Validation of target *CALM2 *and *CD9 *mRNAs by quantitative RT-PCR**. Quantitative RT-PCR using cell lysates, HuR antibody, and IgG1 from RIP-CHIP analysis confirmed results identifying *CALM2 *mRNA **(A) **and *CD9 *mRNA **(B) **as HuR targets. Change in gene expression is represented as fold increase in HuR immunoprecipitation as compared to IgG1. *GAPDH *mRNA was used as an endogenous control. Error bars represent SEM. p value is < 0.005. Experiments were done in triplicate (n = 3).

**Figure 5:**
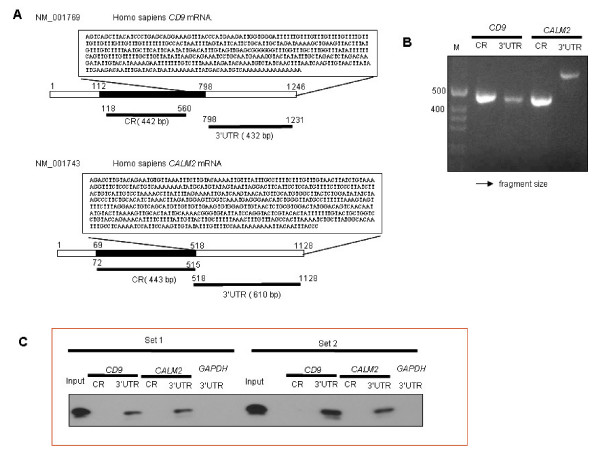
**Biotin Pull-down of *CD9 *and *CALM2*. A**. Scheme of Coding region (CR) and 3'UTR fragments for biotin pull-down assay. The sequences were obtained from Entrez data base. CR and 3'UTR fragments selected for amplification by PCR are as noted. **B**. 1% agarose gel electrophoresis showing PCR amplified products of the coding regions and 3'UTR's for *CD9 *(442 bp and 432 bp, respectively) and *CALM2 *(443 bp and 610 bp, respectively). **C**. Biotin pull-down assay using lysates prepared from MB-231 cells. The binding of HuR to biotinylated 3'UTR transcripts from both *CD9 *and *CALM2 *mRNAs was specific. HuR did not bind a biotinylated control (*GAPDH *3'UTR); and did not bind to biotinylated transcripts spanning the CR of *CD9 *or *CALM2*. Experiments were done in duplicate (n = 2).

In order to validate HuR binding to genes identified in Figure 2, we chose two known cancer associated genes, *CD9 *and *CALM2*, highly expressed in both cell lines. Two independent approaches confirmed the physical interaction between HuR, *CD9 *and *CALM2 *mRNAs. Precipitated mRNA from the RIP-Chip experiments were analyzed by quantitative RT-PCR. Both *CD9 *and *CALM2 *mRNAs were enriched in the HuR RIP by as much as 160 fold (Figures 4A and 4B), but not the isotype control IP. We further confirmed HuR binding to *CD9 *and *CALM2 *mRNAs by biotin pull-downs. The relevant portion of the mRNA was transcribed with biotin tags and incubated with lysates from the two cell lines to probe for interactions with protein. The mixture was then separated by pull-down using streptavidin-coated beads and HuR levels were analyzed by Western blot analysis. As seen in Figure 5, HuR specifically interacts with *CD9 *and *CALM2 *mRNAs in the 3'UTR regions, but not within the coding region (CR) or with a control biotinylated RNA corresponding to the 3'UTR of the housekeeping control *GAPDH *mRNA, which is not a target of HuR.

### HuR differentially regulates CD9 and CALM2 in MB-231 and MCF-7 cell lines

**Figure 6:**
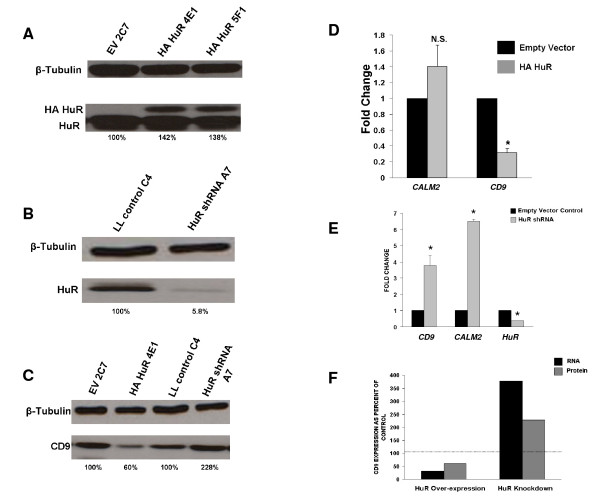
**HuR differentially regulates *CD9 *and *CALM2 *in MB-231. A**. Epitope HA tagged HuR is over-expressed by 142% and 138% respectively, in stably transfected clones 4E1 and 5F1, as compared to empty vector (EV) control clone 2C7. **B**. HuR knock-down using lentiviral short hairpin (sh) RNA H760 results in a 94% reduction in steady state levels of protein in clone A7 (LL = lentilox control). **C**. HuR over-expression results in a 40% reduction in CD9 protein levels as assayed by Western analysis; however, HuR knock-down using lentiviral shRNA results in an increase from 100% to 228% of *CD9 *levels. **D**. Over-expression of HuR decreases *CD9 *mRNA levels but not *CALM2 *expression. Analysis of steady state *CD9 *and *CALM2 *mRNA levels by quantitative RT-PCR reveals significant decreases in *CD9 *mRNA levels, whereas *CALM2 *levels are unaffected. Although *CALM2 *expression appears greater, the change is not significant. **E**. Knocking down HuR levels by shRNA in MB-231 cells shows significant increases in *CD9 *and *CALM2 *mRNA levels by quantitative RT-PCR. Decreased levels of HuR mRNA validate HuR shRNA knock-down. **F**. Graph showing the effects of HuR on the expression of *CD9 *mRNA. HuR over-expression results in decreases in both mRNA and protein levels, though the decreases are greater in RNA. Whereas, HuR knock-down by shRNA results in significant increases at both the mRNA and protein levels, with greater change at transcript levels. The dashed line represents levels in control cells. Error bars represent SEM. p value is < 0.005; N.S. = not statistically significant; * = statistically significant. All experiments were done in triplicate (n = 3).

To gain insight into the biological effects of these associations, we studied the consequences of stably increasing or decreasing HuR abundance. Individual MB-231 clones which over- and under-express HuR were established by limiting dilution (Figure 6A and 6B). MB-231 cells over-expressed HuR by about 140% (Figure 6A). HuR knock-down using lentiviral shRNA resulted in ~95% reduction in HuR expression (Figure 6B). Surprisingly, over-expression of HuR in MB-231 cells caused decreases in both *CD9 *protein and mRNA levels (Figures 6C and 6D). HuR knock-down, however, resulted in increases in both *CD9 *mRNA and protein levels (Figures 6C and 6E). This is the opposite of what we predicted, since HuR is generally regarded as a stabilizer of mRNA. In contrast, over-expression of HuR in MB-231 cells did not significantly alter the levels of *CALM2 *mRNA (Figure 6D). Figure 6F depicts a graphical analysis which reveals that HuR over-expression decreases both *CD9 *mRNA and protein levels, as compared to controls (dashed line set at 100%). Whereas, HuR shRNA knock-down results in increases in both *CD9 *mRNA and protein levels above control levels.

**Figure 7:**
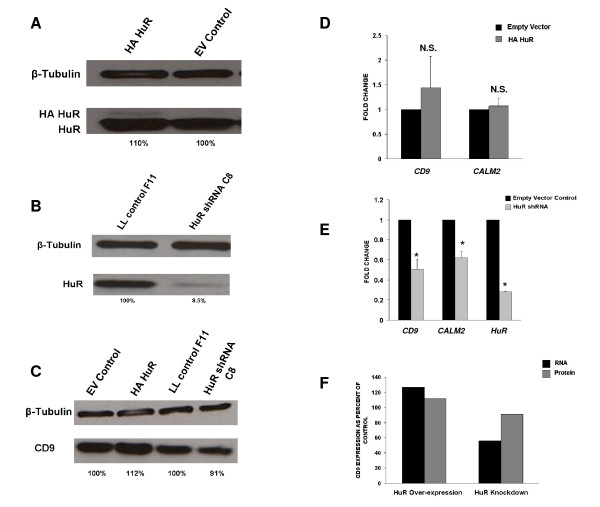
**Effects of over-expressing or reducing HuR on *CD9 *and *CALM2 *expression in MCF-7 cells. A**. Western analysis of HuR over-expression in heterogenous population of cells reveals approximately 10% over-expression. **B**. Lentiviral HuR shRNA efficiently knocks down HuR protein by over 90%. **C**. HuR over- expression and under-expression results in small changes in CD9 protein levels in MCF-7 cells. **D**. Levels of both *CD9 *and *CALM2 *mRNAs are unchanged in cells which over-express HuR; whereas lentiviral knock-down of HuR in MCF-7 cells results in decreases in steady-state mRNA levels **(E)**. The graph in **(F) **shows minimal changes in *CD9 *mRNA and protein levels in HuR over-expressing MCF-7 cells. The *CD9 *mRNA levels, however, are more affected in HuR knock-down. P value is < 0.005; N.S. = not statistically significant; * = statistically significant.

We performed similar analyses with MCF-7 cells, though the over-expression levels of HA HuR were only approximately 10%, since this was a pooled population (we have been unable to obtain MCF-7 clones which over-express HuR). In contrast, we generated MCF-7 clones with reduced HuR levels (93%) using lentiviral shRNA (Figure 7B). Western blot analysis of MCF-7 cells with over-expression of HuR reveals modest increases in *CD9 *protein levels (Figure 7C). There are also modest decreases in *CD9 *protein expression in MCF-7 with reduced HuR levels (Figure 7C). mRNA levels of *CD9 *and *CALM2 *are essentially unchanged in MCF-7 cells which over-express HuR (Figure 7D). As expected, HuR knock-down in MCF-7 cells using lentiviral shRNA resulted in significant reductions in both *CD9 *and *CALM2 *mRNA levels (Figure 7E). The right panel in Figure 7E indicates efficiency of HuR mRNA knock-down which is consistent with the protein data (Figure 7B). These results are summarized in Figure 7F. There are no significant changes seen in *CD9 *mRNA and CD9 protein for HuR over-expression. There is a more pronounced knock-down, however, in *CD9 *mRNA in MCF-7 cells with reduced HuR levels.

The results of HuR shRNA knock-down in MCF-7 cells were as expected, but opposite of those seen for MB-231 cells. Steady-state mRNA levels of *CD9 *and *CALM2 *mRNAs decreased, consistent with the hypothesis that HuR generally stabilizes its mRNA targets. One possible explanation of these disparate results is different levels of total cellular or cytoplasmic HuR. We performed nuclear and cytoplasmic fractionation (Additional File 3, Figure S3). These results demonstrated modest (approximately 10%) greater cytoplasmic levels of HuR in MB-231 cells as compared to MCF-7. The total cellular HuR levels are very similar for both MB-231 and MCF-7 cells. Taken together, these results indicated that HuR appeared to differentially regulate the same mRNAs in a manner dependent upon the cellular milieu.

## Discussion

We utilized RIP-Chip technologies to define differentially regulated HuR genes in ER+ and ER- breast cancer. To our knowledge, this is the first report of a side-by-side genome-wide comparison of HuR-associated targets in wild type ER+ and ER- breast cancer cells. Our findings indicated that HuR interacts with small subsets of genes in breast cancer, out of the possible 8% of human genes possessing AREs which are potential HuR targets. Three broad categories of HuR targets were identified. First, there was a subset of targets only found in ER+ breast cancer. Second, there was a unique subset of HuR targets found only in ER- breast cancer. A third subset consisted of HuR-associated mRNAs common to both forms of breast cancer, many of which were previously described as having roles in cancer.

We selected and validated two HuR targets, *CD9 *and *CALM2 *mRNAs, which were found in high abundance in both types of breast cancer. Initially, we employed the previously developed "heat map" signature of HuR binding to gain insight into putative HuR target sequences [30]. HuR binding was verified by HuR immunoprecipitations, and analyzed by quantitative RT-PCR and biotin pull-downs. Both *CD9 *and *CALM2 *mRNAs were enriched in HuR RIPs compared to isotype control IP reactions. Biotin pull-downs verified the binding of HuR protein specifically to the 3'UTR regions of both mRNAs, as had been predicted. CD9 is a tetraspanin molecule which plays important roles in cellular development, activation, growth and motility. It has been implicated in a variety of cancers, including but not limited to gastric cancers and B cell acute leukemia [58-60].

The role of CALM2 in cancer is less well understood but may be linked to cancer since it is involved in controlling calcium signaling [61,62]. There are three *CALMODULIN *genes (*CALM1, CALM2 *and *CALM3*) highly expressed in both MB-231 and MCF-7 cell lines (Additional File 4, Figure S1). Interestingly, although they are encoded by different genes at different chromosomal locations, all three encode the same open reading frame but differ in the 5' and 3' UTRs [[61,63], and [64]]. Only *CALM2 *mRNA interacts with HuR by RIP analysis. Moreover, previously published reports have indicated the necessity of knocking down all three *CALMODULIN *mRNAs by siRNA to achieve knock-down of the protein [61]. We conclude that there may be differential HuR associated regulation of these *CALMODULIN *genes in breast cancer, even though the mechanism needs to be further delineated.

Surprisingly, the regulation of both *CD9 *and *CALM2 *target genes was dependent upon the cellular milieu. To test the functional consequences of HuR binding to these two transcripts, we prepared cells that stably expressed higher or lower HuR, compared to the parent cells, in both ER+ and ER- breast cancer cell lines. HuR appears to differentially regulate the expression of *CD9 *in opposite directions in the two different forms of breast cancer. Specifically, HuR over-expression in ER- breast cancer (MB-231) paradoxically decreased *CD9 *mRNA and protein levels, whereas HuR knock-down increased the *CD9 *mRNA levels. This is the opposite of what is predicted for most HuR targets, since HuR is thought to stabilize its mRNA targets and often increases their translation. There did not seem to be similar effects upon *CALM2 *expression. As expected, knock-down of HuR by shRNA decreased expression of *CD9 *and *CALM2 *in ER+ breast cancer (MCF-7). Though there are differences in cytoplasmic HuR levels in MB-231 cells as compared with MCF-7, these are modest (10%). This is in keeping, however, with observations that MB-231 cells are more undifferentiated and more aggressive.

Moreover, analysis of HuR-associated mRNAs in both ER+ and ER- breast cancer revealed three broad categories of genes. First, there were well known cancer genes, such as *PTMA*, which are regulated by HuR [27]. Second, there were cancer-related genes, such as *CD9 *and *CALMODULIN*, which were not known to be HuR regulated until this report. Third, there were other genes identified by HuR association with unknown cancer function. These could potentially represent novel cancer targets. Additional proof of HuR involvement with other known cancer genes, such as *CD44 *and *GATA-3*, may represent novel insights into the mechanisms of regulation of these cancer targets (see Additional Files). These results may therefore advance the field by shedding insights into posttranscriptional regulation of known and perhaps unknown cancer target genes.

Though the exact mechanisms of HuR differential regulation of *CD9 *and *CALM2 *are presently unclear, it may involve microRNA (miRNA) regulation. In a recent report, we described the recruitment by HuR of miRNA let-7 to translationally silence *C-MYC *expression [46]. It is clear from the findings of laboratories headed by Filipowicz, Steitz and other investigators, that RBPs and miRNAs are involved in intricate associations to affect downstream translational suppression or activation of target mRNAs to help meet cellular needs [65,66]. Sharp and colleagues proposed that different interactions between RBPs and miRNAs may have evolved as a protective mechanism for the cell against environmental stress [67].

A remaining question is why HuR selectively binds to certain genes containing AREs. Our previous work has demonstrated the role that HuR plays in myogenesis by stabilizing the expression of three critical genes involved in myogenesis: *MYOD*, *MYOGENIN*, and *p21*^*cip*1 ^[68]. HuR over-expression results in precocious muscle differentiation and HuR siRNA knock-down prevents muscle differentiation [69]. It is highly probable that there are more than three HuR targets inside these cells. A specific phenotype potentially arises when HuR levels are altered which may involve interactions with miRNAs, although this theory needs to be fully investigated.

Our findings share some similarity to earlier reports of HuR RIP-Chip analysis of MCF-7 cells stably transfected with MCT-1 [45]. These analyses, however, were not genome-wide and employed transfected cells. Nevertheless, thrombospondin, a known important anti-angiogenic factor, was identified as a HuR-regulated target. Combined with earlier reports of the role of HuR in regulating, VEGF-*α *and HIF1α, HuR may be controlling a "posttranscriptional mini-operon" involved in angiogenesis [29,32,70]. Further studies are being conducted in our laboratory to investigate the role of HuR in breast cancer angiogenesis using xenograft animal models. It will be particularly important to test the role of HuR upon *CD9 *and *CALM2 *expression in breast tumors *in vivo*.

Posttranscriptional gene regulation is increasingly being appreciated as a driver of malignant transformation. The roles of both RBPs and miRNAs (so-called oncomirs) are being recognized in cancer [71]. Many reports have described alterations in miRNA expression profile and function as contributing to breast cancer malignant transformation and metastasis [72-75]. HuR RIP-Chip analysis may shed further light into malignant breast cancer transformation by identifying HuR associated mRNAs.

We believe that there are potential applications for tamoxifen resistance as well. Keen and colleagues have described a potential mechanistic link between HuR expression and tamoxifen drug resistance [76]. As breast cancer cells acquire tamoxifen resistance, there are increased levels of cytoplasmic HuR expression. Increased cytoplasmic HuR levels have previously been described in situations where HuR actively influences expression of cytoplasmic targets [18,47,48]. Drug resistance could be reversed by using siRNA to knock-down HuR expression, whereas exogenous over-expression of HuR could cause cells to become resistant to tamoxifen. We therefore propose that HuR may be coordinately regulating genes which may allow a cell to acquire tamoxifen resistance. It will be interesting to further investigate HuR-associated target genes in ER+ cells in this light.

## Conclusion

In summary, using RIP-Chip analysis, we have performed for the first time a genome-wide comparison of HuR-associated targets in wild type ER+ and ER- breast cancer. We have identified novel HuR targets and have gained insight into the role HuR plays in regulating known cancer genes. We found distinct, differentially expressed subsets of HuR cancer related genes in ER+ and ER- breast cancer cell lines. Based on our observations, the enhanced expression of these mRNA subsets by HuR can influence many of the acquired capabilities of cancer cells. Further investigation into HuR's role in regulating these genes may provide novel insights into breast cancer diagnosis and therapy.

## Abbreviations

(ER-): Estrogen receptor negative; (ER+): estrogen receptor positive; (RIP): RNA immunoprecipitation; (RIP-Chip): RNA immunoprecipitation applied to microarrays; (3' UTR): 3' untranslated region; *ELAV1*: *(embryonic lethal abnormal vision 1*).

## Competing interests

The authors declare that they have no competing interests.

## Authors' contributions

UA supervised the study. RC planned the experiments, performed microarrays and wrote the paper. MMG generated and performed analyses on cell lines as well as quantitative RT-PCR experiments. JWD performed statistical analyses on microarray experiments. JDM assisted in analyses of cell lines. YK and MG planned and performed biotin pull-down experiments. All authors read and approved the final manuscript.

## Appendix

### Microarray Data Preprocessing

Data quality was examined by looking at quality controls metrics produced by Illumina's software (BeadStudio v3.1.3.0, Gene Expression Module 3.2.7). The data were then exported for further analyses in R. Image plots of each array were examined for spatial artifacts, and there was no evidence of systematic effects indicative of technical problems with the arrays. Within limma, quantile normalization was used for between chip normalization. Finally, quality control statistics were computed using a variety of Illumina's internal control probes that are replicated on each array. Any probes which were considered "not detectable" across all samples were excluded from further statistical analyses in order to reduce false positives. The determination of "not detectable" was based upon the BeadStudio computed detection *p*-value being greater than 1%.

### Gene Ontology Gene Universe

In defining the gene universe for the analysis, non-specific filtering was used to increase statistical power without biasing the results. We started with all probes on the Illumina array which had both an Entrez gene identifier [77] and a GO annotation, as provided in the lumiHumanAll.db [78] annotation data package and GO.db [79] annotation maps (built using data obtained from NCBI on 4/2/08). This set was then reduced by excluding probes that exhibited little variability (interquartile range (IQR) of <0.1 on log_2 _scale) across *all *samples because such probes are generally not informative. Finally, for probes that mapped to the same Entrez identifier, a single probe was chosen in order to insure a surjective map from probe IDs to GO categories (via Entrez identifiers). This was necessary to avoid redundantly counting GO categories which produces false positives. Probes with the largest IQR were chosen to be associated with an Entrez identifier.

## Pre-publication history

The pre-publication history for this paper can be accessed here:

http://www.biomedcentral.com/1471-2407/10/126/prepub

## Supplementary Material

Additional file 1 **Figure S2. Table of complete GO analysis**. Listing of HuR-associated genes with odds ratios and functional categories. Click here for file

Additional file 2 **Table S1. HuR targets five fold or greater**. Listing of HuR-associated mRNAs in MB-231 and MCF-7 cell lines. Click here for file

Additional file 3 **Figure S3. Total cellular levels of HuR are similar in MB-231 and MCF-7 cells**. Nuclear and cytoplasmic separation was performed to measure levels of HuR in different compartments of MB-231 and MCF-7 cells. Total cellular HuR levels were very similar, whereas there was a small (10%) increase in HuR cytoplasmic levels in MB-231 cells as compared to MCF-7. Absence of β-tubulin staining demonstrates integrity of isolation as there should not be β-tubulin in the nuclear fraction. Bands were measured by densitometry and normalized to β-tubulin controls. (T = total cellular lysate; C = cytoplasmic lysate, N = nuclear lysate). Click here for file

Additional file 4 **Figure S1. Relative baseline values of *CALM1*, *CALM2*, *CALM3*, and *CD-9 *mRNAs in ER+ and ER- cells**. Quantitative RT-PCR performed on mRNA extracted from cell lysates showing relative levels of *CALM1*, *CALM2*, *CALM3*, and *CD-9 *mRNAs in MB-231 and MCF-7 breast cancer cells. All values were normalized to *GAPDH *mRNA. All experiments were done in triplicate (n = 3) except for *CALM3 *(n = 2). Click here for file
