# Supplementary figures and images for: The RNA binding protein HuR differentially regulates unique subsets of mRNAs in estrogen receptor negative and estrogen receptor positive breast cancer
Source: BMC Cancer. 2010 Apr 6;10:126. doi: 10.1186/1471-2407-10-126 (PMC2856550; doi:10.1186/1471-2407-10-126)

## Slide 1
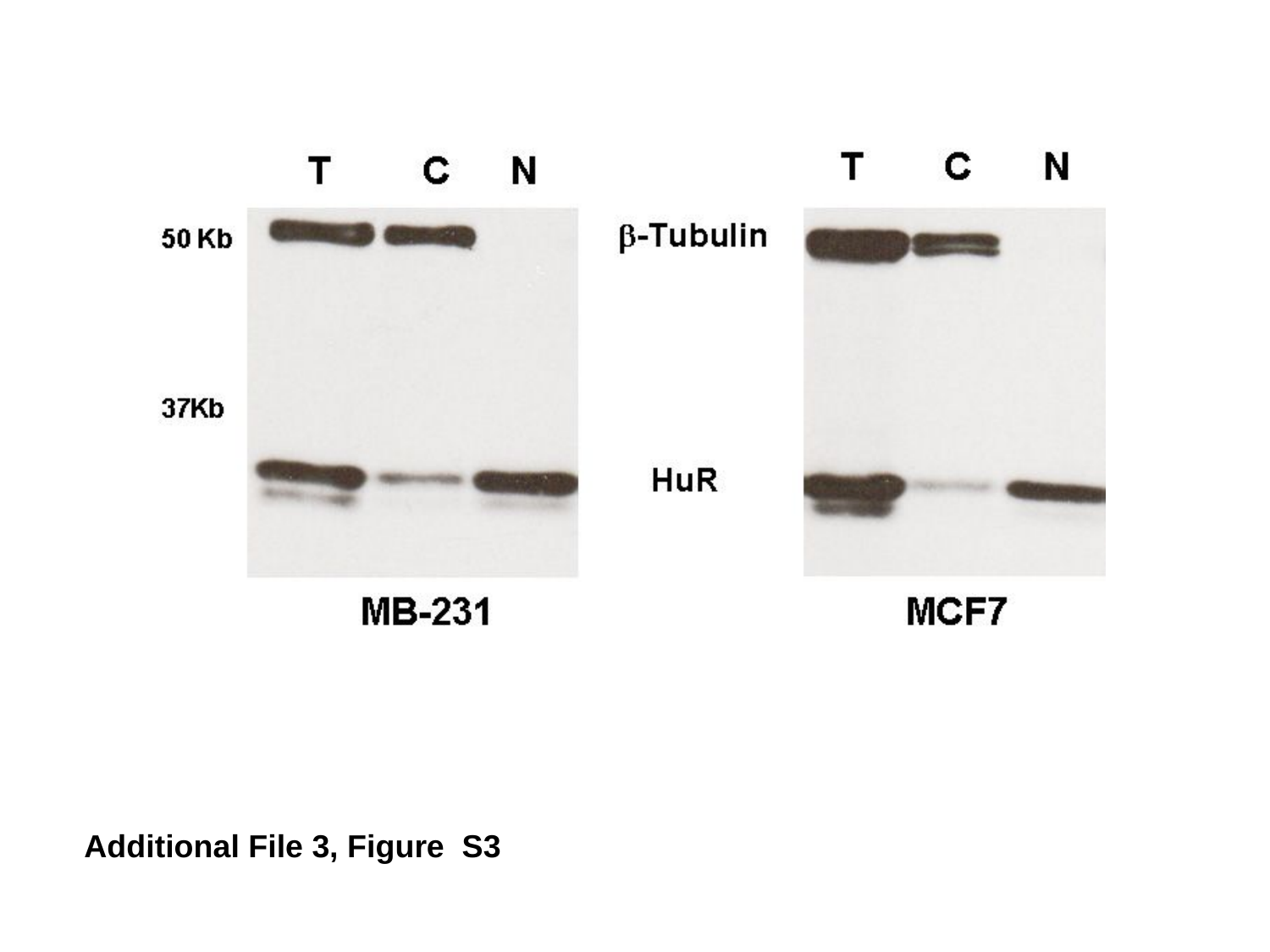

Additional File 3, Figure S3

Supplement: Additional file 3 — Figure S3. Total cellular levels of HuR are similar in MB-231 and MCF-7 cells. Nuclear and cytoplasmic separation was performed to measure levels of HuR in different compartments of MB-231 and MCF-7 cells. Total cellular HuR levels were very similar, whereas there was a small (10%) increase in HuR cytoplasmic levels in MB-231 cells as compared to MCF-7. Absence of β-tubulin staining demonstrates integrity of isolation as there should not be β-tubulin in the nuclear fraction. Bands were measured by densitometry and normalized to β-tubulin controls. (T = total cellular lysate; C = cytoplasmic lysate, N = nuclear lysate). [file 1471-2407-10-126-S3.PPT]

## Slide 1
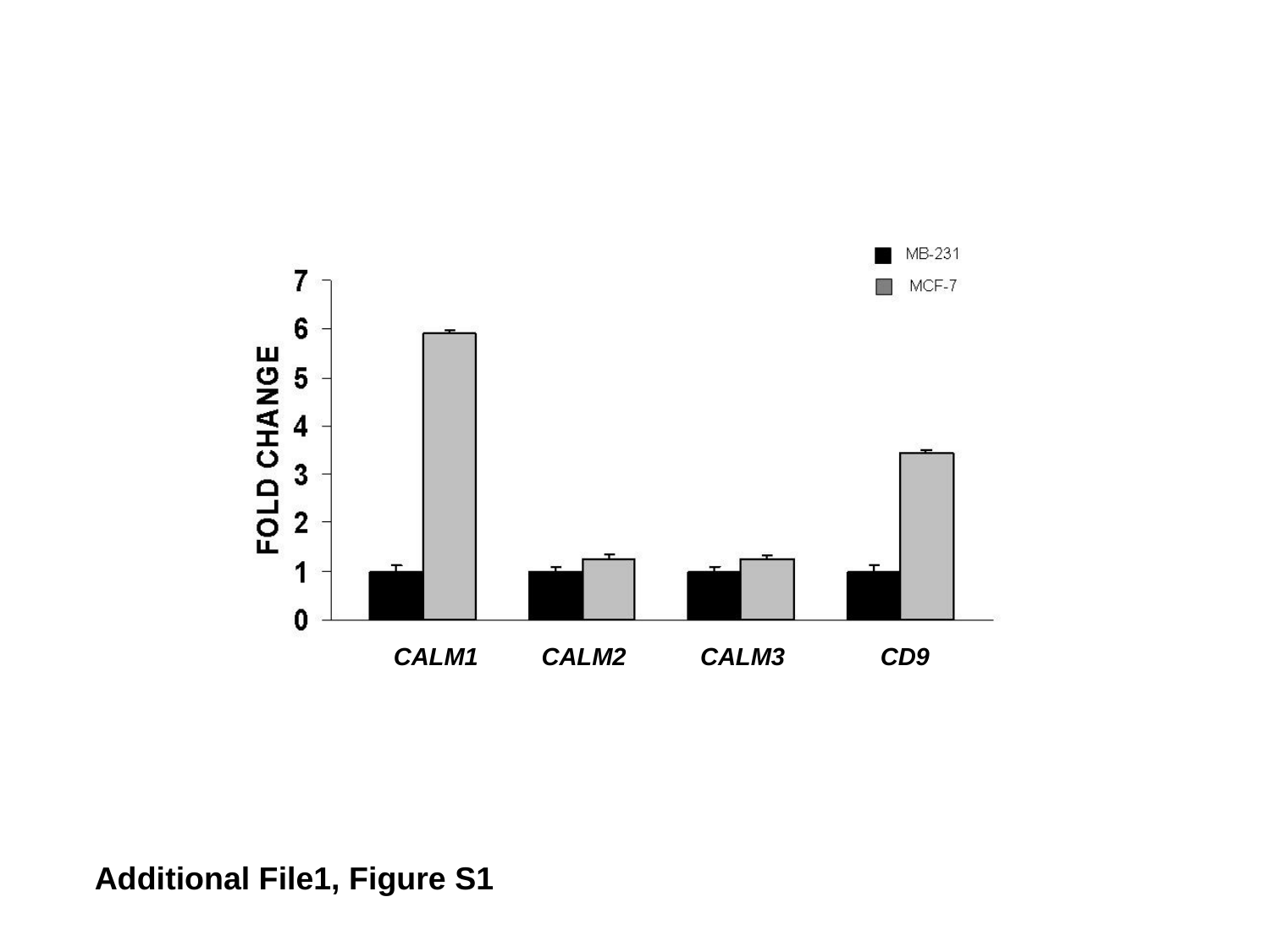

Additional File1, Figure S1
CALM1
CALM2
CALM3
CD9

Supplement: Additional file 4 — Figure S1. Relative baseline values of CALM1, CALM2, CALM3, and CD-9 mRNAs in ER+ and ER- cells. Quantitative RT-PCR performed on mRNA extracted from cell lysates showing relative levels of CALM1, CALM2, CALM3, and CD-9 mRNAs in MB-231 and MCF-7 breast cancer cells. All values were normalized to GAPDH mRNA. All experiments were done in triplicate (n = 3) except for CALM3 (n = 2). [file 1471-2407-10-126-S4.PPT]
